# Supplementary material for: Involving the headteacher in the development of school-based health interventions: A mixed-methods outcome and process evaluation using the RE-AIM framework
Source: PLoS One. 2020 Apr 2;15(4):e0230745. doi: 10.1371/journal.pone.0230745 (PMC7117733; doi:10.1371/journal.pone.0230745)
Supplement: Attachment — Submitted filename: Response to Reviewers.docx [file pone.0230745.s001.docx]

**Response to Reviewers**

Reviewer #1: Lines 72-74 – The Action Schools! BC framework is cited as the example intervention but would be helpful to clarify at first citation or in Table 1 (line 106) if any of the intervention components described in this intervention overlap with or were a featured component from Action Schools! BC. Asked slightly differently, I don't see any of the Action Schools! BC journal citations in the supporting evidence column. Does this mean the components from that intervention are not included in the intervention options for this research project?

***Thank you for highlighting this. It is the choice-based approach of the AS!BC framework that we are incorporating in the CLASP project, as opposed to the individual components themselves, which is why the citations are not references within the supporting evidence column. The following sentence has now been added:***

***“Despite designs such as the AS!BC, there remains a paucity of research where headteachers have complete autonomy over their school’s interventions and the popularity of the choice-based approach of the AS!BC framework warrants further exploration.”*** (Line 75)

Line 85 “Deprivation was classified…” – is this phrase referring to exclusion or inclusion criteria?

***Apologies for the lack of clarity. This isn’t referring to an exclusion or inclusion criteria; the classification was included*** ***to assess the socioeconomic variability of the schools included. We have now clarified this:***

***“Deprivation was classified to assess the socioeconomic variability using individual free school meal entitlement (21) with free school meal eligibility (FSM) ranging from 9% to 53% (mean 37.5%).”*** (Line 91)

Line 106 – there are a number of peer-reviewed journal articles that highlight the impact of classroom-based physical activity. The first reference below would likely inform this article; it is more current than reference 24.:
• Watson A et al. Effect of classroom-based physical activity interventions on academic and physical activity outcomes: a systematic review and meta-analysis. Int J Behav Nutr Phys Act. (2017)
• Carlson JA et al. Contextual factors related to implementation of classroom physical activity breaks. Transl Behav Med. (2017)
• Donnelly JE et al. Classroom-based physical activity, cognition, and academic achievement. Prev Med. (2011)

***Thank you for noting these peer-reviewed articles. These have now been included in the table with reference to classroom-based physical activity and reference 24 has been removed. These journal articles have also been included elsewhere within the discussion where relevant.***

Line 106 - Question – In Table 1, “In The Zone” project, it was noted there was a “training workshop for lunchtime supervisors.” Was teacher training provided for the other 4 interventions? Or, were there "overviews" for teachers regarding how the other 4 interventions should be implemented with fidelity (even if they were led by a coach) to help with sustainability?

***Teacher training was only provided for the “In the Zone” project as this was part of the project itself. Teachers were provided with an overview for the other interventions, though these were relaxed in their instructions to allow the school greater autonomy over how the intervention was delivered. The following sentence has now been added to the ‘Intervention components’ section:***

***“All five were free to the schools and pupils, all costs were covered through CLASP, and teachers were provided with an overview of the how their chosen interventions should be implemented.”*** (Line 111)

Line 112 – are the UK sites operating in a year-round school setting? (Follow-up in July would be almost impossible in the U.S. school systems so indicating the schools operate year-round would help a reader).

***Thank you for highlighting this, which is important for international reach. We have now included that the UK school structure runs from September to July.***

***“For reference, the UK school structure runs from September to July.”*** (Line 122)

Lines 134-135 – RE: “The two other coaches declined invitation to participate in an interview due to work commitments.” Questions: Were these coaches paid by the study to participate as physical activity (PA) leaders only? Were they employed by the school (e.g. as physical education teachers) or external PA leaders/coaches, for example, from a local fitness or recreation center? Was the interview a condition of engagement as a coach at the front end of the study? Did they receive a stipend for “coaching” and providing feedback on the intervention?

***The coaches were paid by the study to participate as external PA leaders only. The interview was not a condition of the employment and*** ***the coaches did not receive any additional funding for taking part in interviews, so as not to influence the involuntary nature of their involvement in this aspect. We have now clarified this with the following sentence:***

***“******No additional funding was provided for their participation in interviews, so as not to incentivise their involvement.”*** (Line 144)

Line 144- 147 – notes pupils were selected randomly and then “Those pupils who did not participate in optional interventions, such as alternative activities, were included in the focus groups to understand reasons underpinning lack of engagement.” Questions: 1) Were those pupils who did not participate in the interventions in the same focus groups as those who did *participate? 2) Did those pupils who did not participate have consent to participate and chose* not to, that is, was participation optional by the teacher?

***Pupils who participated in the physical activity interventions were present in the same focus groups as those who did not in order to promote further discussions and gain a greater understanding from both groups of pupils. This has now been clarified as shown below:***

***“Engagers and non-engagers participated together in the focus groups in order to promote more organic discussions regarding facilitators and barriers. Participants were selected via stratified randomisation to ensure equal numbers.”*** (Line 160)

***Separate consents were required for participation in the research aspects such as focus groups and the physical activity interventions. Only those who had consent to participate in the research aspects were eligible to be involved in the focus groups and pupils were selected at random from those eligible.***

***The following sentence has been added into the ‘Ethical approval’ section, in order to improve clarity:***

***“Written informed parental consent and child assent was obtained prior to participation in the research components (e.g. focus groups). Parental consent forms were also required for participation in afterschool sessions.”*** (Line 103)

Line 233 – Is it possible to include the range of pupils / parents that were reached for the after school activities (even if attendance records were sporadic)? Given intervention strength is often determined by dose x reach this seems to be a disclosure gap.

***Thank you for raising this interesting point. Whilst we agree this would be useful information, there is not enough data to quantify this with any certainty, and attendance was not the main focus of the study. However, we acknowledge that this has not been addressed fully within the manuscript and we have therefore included this as a limitation within both the methodology and the discussion.***

***“Attendance records were completed sporadically, leading to insufficient data capture, and therefore this data could not be quantified with any certainty.”*** (Line 251)

***“Moreover, the sporadic recording of attendance of afterschool sessions prevented reach being calculated with any certainty.”*** (Line 547)

Line 298 - Coach consistency, enthusiasm and session delivery: It would be helpful for a reader to have one or two opening statements that basically summarize what supported implementation and what deterred implementation and or pupil participation.

***Thank you for your suggestion. The following sentence has been added to briefly summarise this section.***

***“The impact of the specific coach, and their approach to the sessions, was highlighted as influential, with enthusiasm, confidence and consistency all key factors in both engaging the pupils and maintaining delivery of the sessions.”*** (Line 313)

Lines 378-379 - it would be helpful to have a sample testimonial statement for this text: "the idea of using daily energisers as an active break when concentration waned appealed as a future approach"

***We appreciate a sample testimonial statement would be helpful in this instance and therefore the following quote has now been added.***

***“However, the teacher stated that the daily energisers would be best used ‘more for concentration I think...especially in primary school they have break time in a morning, they have a break time in the afternoon, and they’re always up on their feet moving about the class, so I don’t feel that it makes a lot of difference to their healthy lifestyle’”.*** (Line 396)

Line 380 - Parental recruitment strategies are not outlined in the paper. Understanding what techniques were used would be great. Were paper notices sent home? Were intervention details (time commitment) sent via email or in a voice mail? Were there reminder notices to parents?

***Prior to the interventions being implemented, parents were invited to a coffee morning at the school to discuss the different activity types they would like to participate in. Once they had agreed, leaflets notifying the days and times of the sessions taking place were sent out to parents and pupils. Building on from the following sentence:***

***“Pupils were administered surveys by researchers prompting selection of varying types of sports or activities, and parents were invited to a coffee morning at the school to discuss different activity types.”*** (Line 236)

***We have therefore provided an additional sentence to provide further detail:***

***“Leaflets notifying the days and times of the sessions taking place were sent out to parents and pupils.”*** (Line 238).

Line 392 - Clarity is needed early in the manuscript re: teacher and pupil input on selection of intervention activities. To what extent did the headteachers consult with / get the opinion of other teachers and pupils when they selected the interventions or did they make the decision on their own? Was the same selection process employed at School A, B and C? Line 403 says "Whilst school staff enjoyed the opportunity to select their own interventions, in some instances there was discordance between pupils’ and headteachers’ tastes." This sentence makes it sound like teachers could pick their own strategy but earlier descriptive text seemed to suggest the headteachers selected the interventions.

***Overall, headteachers, some with input from other teachers, chose which intervention component, then pupils chose the type of intervention, (e.g. street dance, basketball). We believe that the following sentences provide clarity, but if the reviewers have any further specific suggestions, we are willing to consider them.***

***“Although all three schools were provided with autonomy over intervention choice, the three headteachers exercised their autonomy in very different ways and opted for different approaches to tackle their school's physical activity needs (Fig 2). During the initial interviews, headteachers from two schools (A and B) mentioned that they strived to be democratic in their approach and discussed the options with respective deputies or P.E. co-ordinators. However, Headteacher C took a more autocratic approach.”*** (Line 225)

***Following feedback from pupils after the interventions had started, headteachers realised they should have consulted more with pupils in the first instance to ensure what was being delivered was to the pupils’ tastes.***

Line 439 - Encouraging "impact on ...maintenance" seems to be an overstatement given the results reported in the previous section.

***Following comments from yourself and Reviewer 2 to rework the opening paragraph of the discussion, this section has been amended and now reads as follows:***

***“This study aimed to involve headteachers in the developmental stages of school-based health interventions to allow them greater autonomy and explore how this influenced adoption, effectiveness, implementation and maintenance. The CLASP intervention demonstrated that providing headteachers with a choice of physical activity projects was a positive approach to the adoption of a school-based intervention as this was viewed as a more collaborative approach to working. Mixed results were reported for the effectiveness, implementation and maintenance of an autonomous model. However, contributing influential factors were similar to those reported in more traditional school-based health interventions, such as a lack of time and existing curriculum pressures.”*** (Line 458)

Line 502 - Strengths and Limitations: The lack of feedback from coaches at follow-up would seem to be a limitation. The ability of a school to sustain an intervention that requires an external coach would seem to be challenging for schools unless the coach is employed by the school system and paid for his/her time.

***The authors agree that the lack of feedback was a limitation, and this has now been added to the manuscript as follows:***

***“The lack of feedback from all the coaches at follow-up was also a limitation.”*** (Line 546)

***Unfortunately, the requirements of funds are a limitation of all real-world interventions and as no cost-effective analysis was undertaken, due to this being outside of the scope of the study, we felt it was best not to comment on funding requirements at this stage.***

Figure 2 - Level of choice - does the down arrow indicate that all levels between the headteacher and the children had a choice of the intervention? There should be an acronym abbreviation in column School A Intervention chosen to define (Street Dance & Basketball [SD/B])

***Yes, this is correct. The down arrow represents that the headteacher, teacher and children had a choice in the intervention, whereas for the school C, the headteacher themselves made the choice. Figure 2 has now been reworked to improve the clarity here.***

***An acronym abbreviation and additional information have been added to the Figure legend as shown below:***

***“Legend: The down arrow shows where the headteacher, teacher and children had a choice in the intervention, whereas for the school C, the headteacher made the choice. (SD = Street Dance, B = Basketball, FB = Family Boxfit)”*** (Line 222)

Reviewer #2: Review
With choice-based/autonomous participatory models recommended by the Health Promoting Schools literature this research presents an interesting and relevant implementation evaluation of an approach where schools choose relevant evidence-based PA interventions to implement. The analysis uses a well-accepted public health evaluation framework to explore implementation and outcomes and the research is very comprehensive. The manuscript is well written although the complexity of the evaluation and the amount of data makes the discussion challenging. Following are some revisions that I believe will enhance the manuscript. The discussion is a key area for revision.

Introduction
The discussion includes some reference to the health promoting schools framework and approach however the introduction doesn’t. On page four in the introduction where there is discussion about the “increasing autonomy design of AS! BC” and where a lack of research on autonomous designs is highlighted, the section could be strengthened with the evidence about health promoting schools approaches which, at their most pure, are meant to enhance school level autonomy. The evidence on HPS approaches tends to support focused ‘choice-based’ approaches where schools are constrained to issues like physical activity, healthy eating or mental health. I am not suggesting how to approach this but that the section should be enhanced related to the broader literature on autonomous HPS designed interventions in schools. (Lines 444-445 from discussion could be moved into this section).

***Thank you for highlighting this aspect. The following lines have now been added to the introduction in order to address this:***

***“Guidelines for designing complex interventions suggest that permitting schools an element of local adaptation enables interventions to more closely align with their target population (17). Moreover, the Health Promoting Schools agenda recommends allowing schools more choice in creating their own holistic, health-centred environment that endorses their individual values and ethos (18). Despite these guidelines and recommendations, there are few established health interventions which allow headteachers a choice of autonomy on different types of intervention.”*** (Line 70)

Methods
Page 7 Lines 148-151. The approach to talking to engaged and non-engaged students sounds purposive but you state that pupils were randomly selected. How did you ensure that both types of students were present and represented if you used random selection? (perhaps a detail about how balanced randomized selection ended up being in terms of engaging and non-engaging students). I don’t understand randomly selecting in this situation but at least showing the actual situation that occurred through the process to result in both voices being heard and perhaps highlighting in the results how the comments were similar or different across the two types of students?

***Reviewer 1 also alluded to the lack of clarity with regards to how the engaged and non-engaged pupils were integrated into the focus groups. As described earlier, separate consents were required for participation in the research aspects such as focus groups and the physical activity interventions. Only those who had consent to participate in the research aspects were eligible to be involved in the focus groups. These pupils were separated into engaged and non-engaged, and pupils were selected at random from these two groups. The following sentence has been added to the manuscript:***

***“Engagers and non-engagers participated together in the focus groups in order to promote more organic discussions regarding facilitators and barriers. Participants were selected via stratified randomisation to ensure equal numbers.”*** (Line 160)

***Additionally, more clarity has been added throughout the implementation section to clarify whether the opinions were from pupils who had engaged or not. For example:***

***“This competitiveness for after school time was reinforced in school B, as some boys who did not attend mentioned family boxfit clashed with their running club.”*** (Line 367)

Results
Page 11 Line 252 – do you mean across all schools (overall when all three schools were analysed together? As on the previous line you said only School C was significant? These two statements seem to contradict.

***The first sentence refers to the MVPA results between baseline and post-intervention (3 months), whereas the following sentence refers to the MVPA results between baseline and follow-up (6 months).***

Discussion
Overall I like the comprehensive approach to the study but the discussion is the component of the manuscript that is in need of quite a bit of work. It needs to be tightened and focused on the actual aims and results of the study. As it is currently written it is suggesting findings and conclusions that aren’t supported by the data.

1. I think the discussion needs to start out with an introduction that re-highlights the aim, lays out an order to the discussion based on the aim of the study highlighted on Page 4 and also is ordered based on the presentation of the results which were ordered based on RE-AIM. This will help with clarity and focus on the results.

***Thank you for the suggestion. On reflection, we agree that the discussion did need to be more focussed and this section has undergone considerable changes in order to achieve this. An introductory paragraph has now been integrated at the start of the discussion section which reiterates the aims and the key messages:***

***“This study aimed to involve headteachers in the developmental stages of school-based health interventions to allow them greater autonomy and explore how this influenced adoption, effectiveness, implementation and maintenance. The CLASP intervention demonstrated that providing headteachers with a choice of physical activity projects was a positive approach to the adoption of a school-based intervention as this was viewed as a more collaborative approach to working. Mixed results were reported for the effectiveness, implementation and maintenance of an autonomous model. However, contributing influential factors were similar to those reported in more traditional school-based health interventions, such as a lack of time and existing curriculum pressures.”*** (Line 458)

2. Ultimately the introduction should also highlight the big messages (conclusions) that you will provide details about in the discussion e.g. this mixed methods study showed mixed results related to the adoption and implementation of autonomous models and highlighted that autonomous models may be influenced by school factors highlighted previously in the literature. (and reiterate in the conclusions) A key introductory message is about mixed results but that the study provides valuable insights about autonomous approaches that could inform further development and implementation of such approaches. You suggest something like this in the limitations when you highlight that the RE_AIM framework allowed for insights from a ‘proof-of-concept’ perspective.

***Thank you. This has been addressed in the comment above.***

3. The discussion and conclusions need to be more measured and based on the data. I suggest this because the first sentence 437-438 says it was a positive approach but the adoption rates were similar to, or worse than, choice-constrained studies in the literature (so choice didn’t appear to influence adoption), you didn’t really show effectiveness but potential effectiveness (maybe… those results were very difficult to interpret given the lack of information about participating in the interventions so what caused the effect??). However, during the implementation evaluation the principals told you they liked the choice but 2/3 didn’t sustain. These are very mixed results and need to be discussed far more cautiously.

***We appreciate that with the depth of information within the result’s section of the paper, it has been difficult to collate this all fully within the discussion. This has now been expanded upon in order to discuss all components in further detail. A full track change version of the manuscript has also been uploaded detailing all changes.***

4. You talk about local adaptation in the discussion (Lines 445-449) but you have no evidence that adaptation went on – you didn’t set out to study it, you didn’t collect data about adaptation and you haven’t looked into the adaptation literature. I suggest removing any comments about adaptation and how the approach demonstrates adaptation is both desired and warranted. You provided autonomy/choice. That is the focus of the article.

***We have removed any references to adaptation and have reworked this section as follows:***

***“The increased autonomy given to headteachers during this study allowed them to select intervention components that best aligned with their current priorities and personal values; an important guideline for developing complex interventions (17) and a key recommendation of the Health Promoting Schools agenda (18).”*** (Line 470)

5. Overall I would suggest that the discussion needs to be cut back a bit ensuring that you aren’t re-presenting results but discussing the results in the context of the literature. For instance you go school by school discussing individual reasons for adoption where this could be a global statement about the different rationales for adoption and how they are similar or different – then compare this to adoption factors outlined in the literature. I believe Story et al have presented data on administrators perspectives to implementing HPS that might prove useful to compare to.

***Thank you for your suggestion. The discussion has been more condensed in order to be more succinct, whilst discussing the results within the context of the literature, and we now believe the discussion is sufficiently improved. References to Story et al’s work have also been included.***

***“Previous research has discussed the discordance between administrators’ and teachers’ views on health-based interventions and the impacts on implementation (74).”*** (Line 521)

6. I think both the introduction and conclusion should talk about the key messages – e.g. use of mixed methods and the strength in elucidating the complexity of adoption and implementation. I believe that what you have really started to show is that adoption and implementation autonomous or non-autonomous models may be constrained by similar factors that influence adoption and implementation and are common to the broader school context. This is a very important finding, not yet discussed in the literature.

***Thank you for highlighting this aspect. The discussion has been reworked to hopefully highlight the key messages more clearly. For example, the following section has been included:***

***“Afterschool sessions proved popular from a headteacher perspective, as they were less burdensome on schools in terms of implementation. Time and curriculum pressures have regularly been identified as a barrier to the implementation of traditional school-based physical activity initiatives (15, 68) and this does not appear to be specific to an autonomous approach.”*** (Line 479)

7. Effectiveness results need to be discussed in light of reach. If you have a completely voluntary intervention in two schools but see increases in physical activity across a set of children that volunteer with no control group are you seeing an intervention effect? If it is cross-site comparison it is no surprise that the universal strategy showed a significant outcome in terms of MVPA but the sedentary results are very hard to explain. How are these children less sedentary when no intervention focused on this and only one showed differences in MVPA (did your LPA show changes?)? Is this seasonality? These data have significant limitations that are not even discussed in the limitations section. They need to be.

***Thank you for your insight regarding the physical activity results. We have made the following changes and this section now reads as follows:***

***“Whilst all three intervention choices reported favourable changes to MVPA, sedentary time and fitness, these results should be interpreted with caution. Given the timing of the intervention (January through to July), the influence of seasonal variation*** ***(Winter to Summer) cannot be precluded (76). Additionally, school A mentioned that participating in this study increased awareness of pupils’ physical activity levels, and the*** ***impact of additional school interventions cannot be separated out. The lack of feedback from all the coaches at follow-up was also a limitation. Moreover, the sporadic recording of attendance of afterschool sessions prevented reach being calculated with any certainty. Future studies would benefit from the use of age- and sex-matched comparison schools to provide additional insights into the results reported here.”*** (Line 541)

Limitations
There is a body of implementation literature that shows Principal support is important and choice is important but a head teacher is one individual that works with a number of other professionals in a school to implement. In some jurisdictions the equivalent of the Head teacher doesn’t adopt anything without asking the teachers if they are willing. The limitations of the administrators choosing need to be discussed. There was a theme where one school did take a ‘consultative approach with their staff’.

***Thank you for your suggestion. This element of autonomy has now been explored in the discussion and the implications of including teachers and pupils has been explored. For example:***

***“Interestingly, only one headteacher fully discussed the intervention option with their teachers before implementation. Whilst it is important to engage the headteachers initially, buy-in is also required across the whole school. Previous research has discussed the discordance between administrators’ and teachers’ views on health-based interventions and the impacts on implementation (74). Moreover, a supportive school climate has been identified as a key factor for effective implementation (68).”*** (Line 518)

Page 20 and 21 Lines 460-461 – this head teacher’s opinion is supported by Geoffrey Rose and the population health concept that universal strategies that create small changes in behavior are more beneficial at the population health outcome level than interventions targeted at those at highest risk. You could add a comment to this effect. However, above I have recommended talking more succinctly and globally about the different rationale for adoption and how interventions may need to offer multiple reasons to Principals to encourage adoption depending on their context and beliefs and that other factors such as staff transitions, illness etc. influenced adoption.

***Thank you for this insight. We have looked into Geoffrey Rose’s work and appreciate you highlighting this. This has now been integrated succinctly within the discussion with reference to the headteacher’s opinion.***

***“Therefore, the headteacher believed they would be improving health and academic achievement for the class as a whole, rather than only the few; a universal approach consistent with the population strategy detailed by Geoffrey Rose (72).”*** (Line 493)

Line 452 –systematic reviews on PA implementation in schools show that curriculum pressure and competing demands are key barriers to school PA implementation. When you are discussing the factors that guided what intervention was adopted it should be placed in the context of that literature. The fact that two of three strategies selected were based on avoiding these barriers and required an external coach is critical. Thus… it may be that when there are no policy drivers a majority of school administrators will select an option that is potentially not as effective nor guaranteed to reach the whole school body, negating the power of the universal school-based approach. In fact your evidence further convinces me of the need for policy interventions accompanied by constrained autonomy interventions and capacity-building to ensure school PA delivery. I don’t expect you to go out on a limb and say this but I am just providing an alternative interpretation of your data to perhaps highlight the need for a more measured interpretation.

***Thank you. This is a really interesting concept that hadn’t previously been considered. We have taken this on board and have discussed this further within the discussion.***

***“This difference in approaches suggests that when offered a choice, headteachers may prioritise interventions which fit best around existing pressures, such as curriculum pressure, as opposed to those which demonstrate greater effectiveness but are more burdensome for schools to implement. Therefore, it is advisable to offer whole school, evidence-based choices which have shown potential for effectiveness, ideally in partnership with capacity-building for schools to aid delivery.”*** (Line 496)

Page 23 Lines 518 – involving pupils in the decision-making process, etc. were not (based on your data) key factors in improving adoption and maintenance. They were key factors suggested by stakeholders as potentially contributing to adoption and maintenance. As well, do you actually mean contributing to implementation and maintenance in this sentence? Adoption is the school adopting the autonomous approach (participating) or the head teacher adopting a certain strategy. It is important to be consistent with your language across the document.

***Thank you for these comments. We appreciate the confusion here and have since amended the wording to be more consistent. The full conclusion in its entirety can be read underneath the comment below.***

Conclusions
I feel that the conclusions currently misrepresent your findings. These need re-writing once the discussion is re-focused and the new approach to the discussing the results is adopted. These should mirror key statements made in the introduction to the discussion and key rationale from the introduction/aims but perhaps offer a recommendation as well (more research needed comparing two approaches etc.)

***The conclusion has been reworded as follows:***

***“******Headteachers perceived that being provided greater autonomy resulted in much more of a partnership approach to school-based interventions and welcomed the idea for future interventions. However, mixed results were reported for the effectiveness, implementation and maintenance of the interventions. Nonetheless, headteachers highlight that involving pupils in the decision-making process and promoting a positive school environment were key factors for enhancing engagement. Promoting inclusive physical activity projects, with a consideration of existing curriculum pressures, aided implementation for headteachers. Overall, this mixed-methods study provides valuable insights about autonomous approaches that could inform further development, implementation and maintenance for future interventions.”*** (Line 556)

Perhaps the second sentence on line 517 “However, whilst this aspect engaged headteachers (did it? 30% of those asked participated – How about “whilst headteachers in this study supported the approach”) it was not solely sufficient to support maintenance of the PA strategy chosen nor did it guarantee effective implementation. This may be because teachers who largely are responsible for implementation were not engaged? or… because larger drivers influence adoption and implementation. It appears that autonomous and non-autonomous models may be influenced by similar implementation factors.

***We have taken on the comments here with regards to the conclusions and this sentence has now been reworded (see above).***

***The point you made regarding the similarities between autonomous and non-autonomous models was also of note and this has been integrated into the discussion. One example of this is the inclusion of the following sentence:***

***“Time and curriculum pressures have regularly been identified as a barrier to the implementation of traditional school-based physical activity initiatives (15, 68) and this does not appear to be specific to an autonomous approach.”*** (Line 481)

I leave it to you to decide on what the key messages are but I do believe that the reframing I suggest makes this a very nice addition to the literature.

***Thank you for your comments.***
